# Towards clinical application of GlycA and GlycB for early detection of inflammation associated with (pre)diabetes and cardiovascular disease: recent evidence and updates

**DOI:** 10.1186/s12950-023-00358-7

**Published:** 2023-10-09

**Authors:** Erik Fung, Eunice Y. S. Chan, Kwan Hung Ng, Ka Man Yu, Huijun Li, Yulan Wang

**Affiliations:** 1 Department of Medicine & Therapeutics, Laboratory for Heart Failure + Circulation Research, Li Ka Shing Institute of Health Sciences, and Centre for Cardiovascular Genomics & Medicine, Faculty of Medicine, The Chinese University of Hong Kong, Shatin, New Territories, Hong Kong SAR, China; 2 Hong Kong Hub of Paediatric Excellence, The Chinese University of Hong Kong, Hong Kong Children’s Hospital, Kowloon Bay, Kowloon, Hong Kong SAR, China; 3 Neural, Vascular, and Metabolic Biology Programme, and Ministry of Education Laboratory for Regenerative Medicine, School of Biomedical Sciences, Faculty of Medicine, Lo Kwee-Seong Integrated Biomedical Sciences Building, Area 39, The Chinese University of Hong Kong, Shatin, New Territories, Hong Kong SAR, China; 4 Department of Epidemiology & Biostatistics, School of Public Health, St Mary’s Campus, Imperial College London, London, UK; 5 School of Medicine, The Chinese University of Hong Kong, Shenzhen, China; 6 Prince of Wales Hospital, Room 124010, 10/F, LCWCSB, 30-32 Ngan Shing Street, Shatin, New Territories, Hong Kong SAR, China; 7 Singapore Phenome Centre, Lee Kong Chian School of Medicine, Nanyang Technological University, Singapore, Singapore

**Keywords:** Glycoproteins, Nuclear magnetic resonance spectroscopy, Inflammation, Diabetes mellitus, Atherosclerosis, Cardiovascular disease

## Abstract

Cardiometabolic diseases are associated with low-grade inflammation early in life and persists into old age. The long latency period presents opportunities for early detection, lifestyle modification and intervention. However, the performance of conventional biomarker assays to detect low-grade inflammation has been variable, particularly for early-stage cardiometabolic disorder including prediabetes and subclinical atherosclerotic vascular inflammation. During the last decade, the application of nuclear magnetic resonance (NMR) spectroscopy for metabolic profiling of biofluids in translational and epidemiological research has advanced to a stage approaching clinical application. Proton (^1^H)-NMR profiling induces no destructible physical changes to specimens, and generates quantitative signals from deconvoluted spectra that are highly repeatable and reproducible. Apart from quantitative analysis of amino acids, lipids/lipoproteins, metabolic intermediates and small proteins, ^1^H-NMR technology is unique in being able to detect composite signals of acute-phase and low-grade inflammation indicated by glycosylated acetyls (GlycA) and *N*-acetylneuraminic acid (sialic acid) moieties (GlycB). Different from conventional immunoassays that target epitopes and are susceptible to conformational variation in protein structure and binding, GlycA and GlycB signals are stable over time, and maybe complementary as well as superior to high-sensitivity C-reactive protein and other inflammatory cytokines. Here we review the physicochemical principles behind ^1^H-NMR profiling of GlycA and GlycB, and the available evidence supporting their potential clinical application for the prediction of incident (pre)diabetes, cardiovascular disease, and adverse outcomes.

**Supplementary Information:**

The online version contains supplementary material available at 10.1186/s12950-023-00358-7.

## Introduction

Recent estimates from the International Diabetes Federation indicate that approximately 1 in 10 individuals aged 20–79 years are living with diabetes worldwide [1]. In older adults (65–99 years old), the proportion of the afflicted has doubled to 1 in 5 [2]. Importantly, cardiovascular disease (CVD) complications including acute myocardial infarction (MI), stroke and heart failure (HF) remain the leading causes of death in patients with diabetes [3–5]. With global aging and an epidemiological transition to increasing incident diabetes in older adults, [2, 5] there is a growing impetus to shift CVD management from intervention of complications to early detection, lifestyle modification and treatment of cardiometabolic disorder.

Diabetic and atherosclerotic CVD are characterized by a long subclinical phase before manifestation of complications. This long latency period presents opportunities for detecting and modifying risks [6]. Recent studies have shown that an abnormal metabolome in childhood indicated by inflammation and dyslipidemia was associated with increased risks of cardiometabolic disease in adulthood and old age [7]. While conventional multifactorial indicators including low-density lipoprotein (LDL) and high-density lipoprotein (HDL)-cholesterol, and C-reactive protein (CRP) can provide useful estimates of future CVD risks, high-field proton (^1^H)-nuclear magnetic resonance (NMR) spectroscopy has emerged as a powerful modality for quantitative profiling of endogenous biochemical substances, lipids/cholesterol, small proteins and amino acids that are associated with cardiometabolic disease risks.

^1^H-NMR can provide reliable profiling of inflammation-modified glycoprotein levels as well as detailed characterization of lipid, cholesterol and lipoprotein concentrations, ratios and subfractions with high accuracy and repeatability (i.e. intra-assay percent coefficient of variation (%CV) < 5%). Each assay requires less than 1 ml of blood plasma or serum to reveal tens to hundreds of target features in the specimen [8]. Within the scope of this review article, we 1) focus our discussion on the composite NMR signals of glycoprotein acetyls (GlycA) and *N*-acetylneuraminic acid (GlycB) as indicators of low-grade inflammation that may be superior to the well-established biomarker, high-sensitivity C-reactive protein (CRP), and 2) appraise their capacity as independent predictors of cardiometabolic disorder, (pre)diabetes, CVD, and adverse clinical outcomes.

## GlycA and GlycB: composite NMR biosignatures of low-grade inflammation

Diabetes and CVD share pathophysiologic features that are linked via endothelial dysfunction, leukocyte activation, enhanced thrombosis and systemic vascular inflammation [9–15]. During acute and chronic inflammation, acute-phase proteins are released by the liver and other tissues into the bloodstream to modulate immune responses (e.g. CRP binding to Fc receptors on immune cells) [16]. These protein structures are posttranslationally modified by glycosylation and acetylation resulting in altered ligand-receptor binding, cell–cell signaling, and interaction with the tissue microenvironment [17]. Underscoring the importance of glycobiology in maintenance of homoestasis, every cell in the body and an estimated 70% of the human plasma proteome bears a glycan structure modifiable by acetylation and sialylation [18, 19].

The quantification of altered functional moieties in glycoproteins is possible with recent advancements in NMR technology and pipeline. NMR is a nondestructive method to analyze specimens with high stability; typically, %CV of 1–5% on repeat measurements are observed between laboratories. The method can achieve high throughput and is able to acquire a spectrum under 10 min per sample on a standard 600 MHz (11.4 Tesla) ^1^H-NMR platform. Using proton (^1^H)-NMR to profile the systemic landscape for inflammation-modified protein structures, this approach has advantages over conventional immunoassays that target a single protein and are affected by variability in epitope binding and signal detection via colorimetric, fluorescent or luminescent methods. Whereas profiling of an individual protein or cytokine can inform about disease mechanisms and pathways in a reductionist manner, holistic detection of low-grade inflammation in the circulation has the advantage of leveraging wide distribution of modifiable protein structures as a sentinel network in the internal milieu.

It is noteworthy that different vendors or platforms may use different deconvolution methods and protocols to derive quantitative information for data analysis. In principle, the final quantitative results are similar but the methodology may differ. For instance, Nightingale Health (Helsinki, Finland) uses a curve fitting model with Lorentzian functions and an in-house algorithm for deconvoluting the signals with optimized parameters [20]. Among three molecular windows (designated LIPO, LMWM, and LIPID) for analysis of NMR spectrum in that pipeline (including 500 MHz and 600 MHz instruments), [8] the first two windows are used for visualising the peaks of GlycA. Nightingale only provides GlycA in their reporting, whereas the Bruker IVDr pipeline (Bruker Biospin GmbH, Ettlingen, Germany) provides both GlycA and GlycB values calculated from integral signals by superimposition of terminal N-acetyl signals (δ 2.03) and branched-chain N-acetyl signals (δ 2.07), respectively, determined by the DIffusional and Relaxation Editing (DIRE) spectra [21]. The Lifespin Bloodscanner profiling system (Lifespin GmbH, Regensburg, Germany) also provides GlycA and GlycB data using proprietary deconvolution algorithms. Most contemporary methods and quantitative profiling protocols are derived from 400–600 MHz NMR instruments. From our experience, the resolution can differ between the instruments; however, concentrations measured should be comparable given long enough recycle delay for conducting the experiment.

**Fig. 1:**
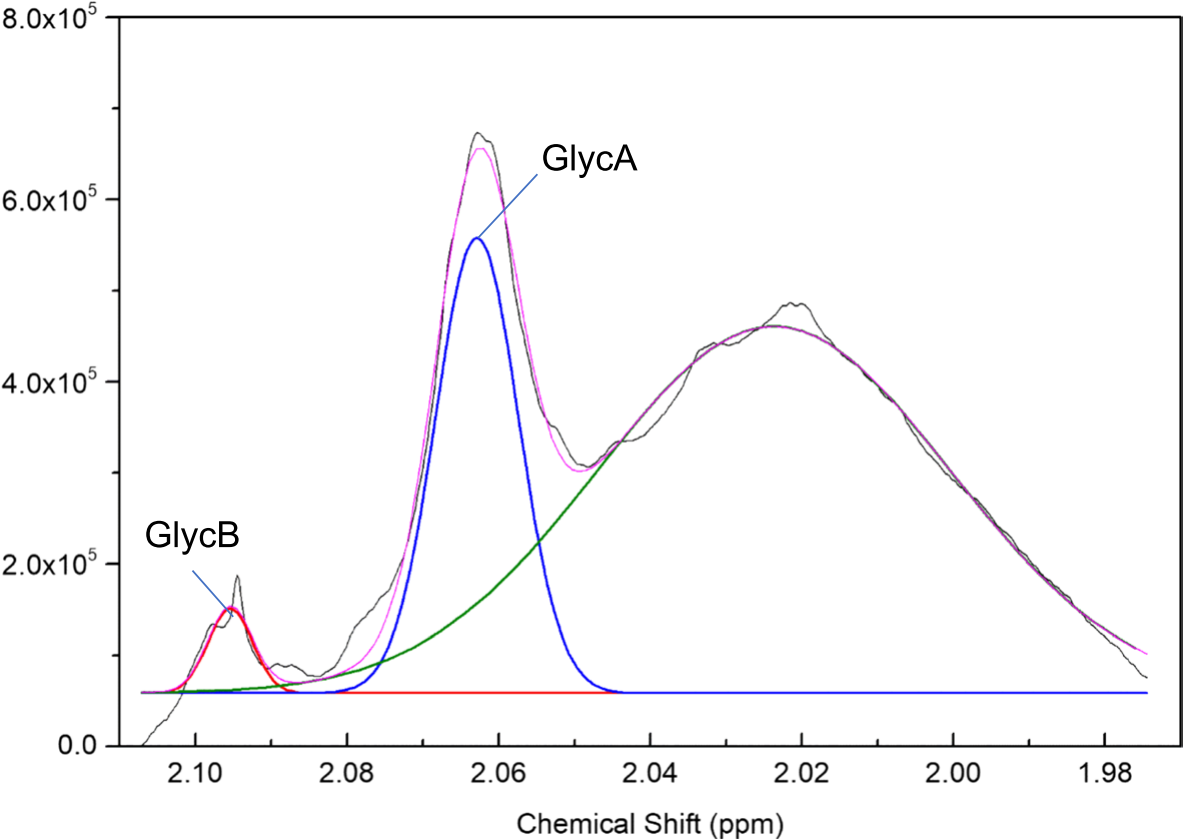
Typical NMR spectrum of glycoprotein region. Black line: NMR spectrum; red, blue and green lines: GlycB, GlycA and lipid peaks respectively generated by deconvolution. ppm, parts per million

GlycA and GlycB are two parts of the glycoprotein signal on the ^1^H-NMR spectrum that are from the same global biochemical process. Bell et al. were first to identify two broad resonance signals centered at δ2.04 and δ2.08 in 500 MHz ^1^H-NMR spectra associated with *N*-acetyl group, primarily *N*-acetylglucosamine and *N*-acetylneuraminic acid, respectively, that were components of acute-phase glycoproteins in human blood plasma [22] (Fig. 1). The composite ^1^H-NMR signals of *N*-acetylglucosamine and *N*-acetylneuraminic acid were subsequently named GlycA and GlycB [23–26]. Specifically, the GlycA NMR signal comprises α_1_-acid glycoprotein, galactosamine, haptoglobin, α_1_-antitrypsin and α_1_-antichymotrypsin, among others, whereas the GlycB signal is primarily contributed by the protons of the 5-*N*-acetyl methyl groups of *N*-acetylneuraminic acid (sialic acid) in glycoproteins [23–27] (Fig. 1 & Supplementary Figure 1); however, the latter is not well characterized. Otvos et al. have documented the identity of GlycB in a U.S. patent published in 2017 (Supplementary Figure 1) [26]. Recognizing GlycB as a distinct inflammatory signal, Fuertes-Martín et al. have shown different methods by which GlycB can be analyzed relative to GlycA [23, 24]. On a deeper level, the characterization and quantification of GlycA and GlycB will depend on the deconvolution models, protocols and methods, as well as the temperature of the test sample [26]. Recently, Noel et al. found evidence linking the GlycA NMR signal to *N*-glycan branching, blood copper concentration, and a single nucleotide polymorphism (rs13107325) in *SLC39A8* (a gene that encodes the cation/bicarbonate symporter protein ZIP8), [28] encouraging further studies to validate associations between NMR inflammatory signals and etiological and pathophysiological factors.

The association of GlycA with chronic inflammation and cytokine networks was first demonstrated by Ritchie et al. using three large-scale cohorts and data from multiple follow-up timepoints in the Cardiovascular Risk in Young Finns Study [29]. The investigators revealed that within-individual elevation of GlycA levels were stable for up to a decade and its elevation predicted long-term risks of hospitalization and death from infections [29]. In sterile inflammation, elevated GlycA has been associated with increased levels of interleukin-6 (IL-6) [25] and TNF-α, [24, 30] among others [29]. Although very few studies have compared GlycA with erythrocyte sedimentation rate, a moderate correlation (Spearman’s correlation coefficient (rho) ~ 0.6–0.7) has been shown in patients with rheumatoid arthritis [31, 32].

GlycA levels are positively and moderately correlated with those of high-sensitivity C-reactive protein (hsCRP), with a consistent correlation coefficient (*r*) of approximately 0.6 [25, 27, 33–35]. Analysis of inflammatory cytokines (e.g. IL-1β, IL-6, IL-10, IL-12, TNF-α) following in vitro stimulation of whole blood with pathogen-associated molecular pattern molecules (e.g. lipopolysaccharide, peptidoglycan) showed a similar direction and pattern of association between inflammatory cytokines and GlycA or hsCRP [36]. Among 36 serum cytokines and hsCRP analyzed in 2,200 participants of the Cardiovascular Risk in Young Finns Study, hsCRP was found to be most strongly associated with GlycA [29].

An advantage of ^1^H-NMR profiling of inflammation over immunoassaying of hsCRP concentration is the markedly lower %CV with the former. Otvos et al. pointed out the relatively high intraindividual variability of assaying hsCRP, as shown in a study of healthy volunteers assessed weekly for 5 weeks in whom %CV was 29.2%, whereas the intra-individual, intra-assay and inter-assay %CV values for GlycA were 4.3%, 1.9% and 2.6%, respectively [25]. This level of assay stability and reproducibility is necessary for longitudinal monitoring and tracking of low-grade inflammation in prediabetes or subclinical vascular inflammation. Independent studies confirmed that intraindividual levels of GlycA were relatively stable over a decade [29, 37].

In exploring the effects of food intake on the body’s inflammatory response, Mazidi et al. found that GlycA levels could fluctuate and reflect what was termed meal-induced inflammation [38]. It was noted that 6 h after food intake, GlycA and IL-6 levels increased by 11% and 190%, respectively, in 60% and 94% of study participants [38]. The study highlighted the varying impact of diet, nutrition and gut microbiota on the circulating metabolome, metabolic variability and immune response. Separate studies have shown that diet can impact cardiometabolic risks and be reflected in levels of GlycA, GlycB, trimethylamine *N*-oxide (e.g. from red meat and dairy products), proinflammatory cytokines and NMR lipoprotein subfractions [39–43]. Compared with GlycA, GlycB was less strongly correlated with CRP levels (typically, *r* = 0.4–0.5) [27]. GlycA and GlycB may differentially provide subphenotypic information on inflammation and adverse clinical outcomes associated with CVD [44].

## Prediction of incident diabetes and prediabetes

GlycA has been shown to predict incident T2D in several large-scale studies [45–47]. In addition to *N*-acetylglucosamine, other acute-phase proteins identified from the NMR GlycA signal included α_1_-acid glycoprotein, α_1_-antitrypsin and transferrin that have been separately reported in T2D [48–52]. In an analysis of 26,508 apparently healthy individuals in the Women’s Health Study who were free of diabetes at study entry and among whom 2,087 (7.8%) developed T2D during a median follow-up of 17.2 years, elevated GlycA was strongly associated with graded increases in the risk of incident T2D as for hsCRP [45]. However, in the PREVEND study, [46] GlycA but not hsCRP was an independent predictor of incident T2D (highest vs. lowest quartile of GlycA, hazard ratio (HR) 1.77 [1.10–2.86]) [46]. Interestingly, the association of GlycA with incident T2D was significant even among individuals with baseline HbA1c < 5%, [45] suggesting that GlycA could be an early inflammatory biosignature prior to the development of prediabetes. In an analysis of circulating metabolome in childhood to predict cardiometabolic risks in adults, GlycA was consistently selected by machine learning as a predictor [7]. In a separate study of obese adolescents with prediabetes who had undergone lifestyle intervention, GlycA levels decreased in parallel with body mass index (BMI), total cholesterol, and 2-h glucose tolerance test values [53].

In the Insulin Resistance Atherosclerosis Study (IRAS) of glycemia, insulin resistance, and insulin secretion involving 1,225 multiethnic participants with or without diabetes, positive correlations were observed between inflammatory markers — GlycA and GlycB (*r* = 0.74), GlycA and hsCRP (*r* = 0.60), and GlycB and hsCRP (*r* = 0.46) (all *p* < 0.001) — that differentially stratified more advanced disease (i.e. impaired glucose tolerance and T2D) from normal or a susceptible clinical phenotype (i.e. normal glucose response and isolated impaired fasting glucose) [27]. All three inflammatory markers were significantly associated with insulin sensitivity index and BMI, but not acute insulin response [27]. However, a separate study found that GlycA did not vary according to the category of glucose tolerance but was instead associated with adiponectin and adipose tissue-related inflammation [54]. Moreover, GlycA was not significantly associated with glucose or HbA1c at the time of hospital admission nor with glucose control. In the Metabolic Syndrome in Men (METSIM) study including 5,401 Finnish men with no history of T2D who were followed for a median of 6.8 years, GlycA was associated with impaired insulin secretion, hyperglycemia and incident T2D, whereas IL-1 receptor antagonist and hsCRP were associated with reduced insulin sensitivity and increased total mortality [55]. Collectively, GlycA has the capacity to indicate systemic inflammation linked to abnormal insulin biology even preceding early diabetes. Further studies are needed to characterize the mechanisms of disease evolution from the early stages of GlycA elevation to development of prediabetes and diabetes.

## Prediction of incident CVD

The evidence base for GlycA as an indicator of atherosclerosis is not incontrovertible, depending on the lesion characteristics under study, the disease stage, and the patient population. A Dutch study did not find a significant association between carotid artery intima medial thickness and GlycA or hsCRP, [56] whereas a study on Spanish T1D patients not on lipid-lowering therapy found significant subclinical carotid atherosclerosis (intima-media thickness > 1.5 mm) and increased levels of GlycA and GlycB, but not hsCRP [57]. In a prospective study of patients with established T2D who were followed up for a median of 3.2 years, 49 of 145 (33.8%) patients developed microvascular complications (i.e. nephropathy, retinopathy, or neuropathy) [58]. Whereas hsCRP was not significantly associated with incident microvascular complications, GlycA was determined to be an independent predictor in multivariable Cox regression models [58]. For each standard deviation above the mean, the risk of incident microvascular complications was increased by 79% even after adjusting for hsCRP [58].

In patients with T2D and atherogenic dyslipidemia (defined as triglyceride > 150 mg/dL [> 1.69 mmol/L]; HDL cholesterol < 40 mg/dL [< 1.03 mmol/L] and < 50 mg/dL [< 1.29 mmol/L] in men and women, respectively), GlycA and GlycB levels significantly improved the discrimination between disease (either dyslipidemia or T2D) and non-disease when added to hsCRP in receiver operating characteristic (ROC) curve analysis [59]. In high-risk T2D patients with coronary and peripheral artery disease, GlycA levels were significantly higher than in their counterparts without peripheral artery disease, and predicted long-term all-cause (but not cardiovascular) mortality [60]. Other prospective studies including T2D and non-T2D patients found elevated GlycA levels to be predictive of incident cardiovascular events [33, 61]. In one study, it was determined that each standard deviation increment in GlycA was associated with a 43% increase in the risk of cardiovascular mortality at 5 years [34].

Further insight into early prediction of cardiometabolic risks has been gleaned from longitudinal follow-up studies of the young and the aged. In a combined analysis of 3,306 adolescents and young adults from the Avon Longitudinal Study of Parents and Children, and the Cardiovascular Risk in Young Finns Study, increased GlycA level was found to be associated with multiple lifestyle-related CVD risk factors, cardiometabolic risks, and vascular dysfunction; whereas associations with hsCRP were driven by BMI with little relationship to cardiovascular risk factors or CVD phenotypes [62]. Although one study found neither GlycA nor CRP in childhood to be predictive of mid-life CVD, [63] a multi-cohort study using machine learning procedures have agnostically selected GlycA along with ApoB-to-ApoA ratio and large HDL-phospholipids in the development of a multifactorial risk score for cardiometabolic risk prediction [7].

GlycA may also be informative for stratifying high-risk patients referred for angiography. Among 900 high-risk Finnish patients who underwent coronary angiography, GlycA in the fourth and fifth quintiles were associated with markedly elevated 12-year risk for incident mortality (HR 4.87 [95% CI, 2.45–9.65] and 5.00 [2.38–10.48], respectively) [64]. In another study of 2,996 patients undergoing coronary angiography, GlycA and hsCRP were independent and additive predictors of future cardiovascular events [65].

## Association with subclinical myocardial dysfunction and heart failure

The proinflammatory effects and nature of HF have long been recognized [66–71]. In HF with preserved ejection fraction (HFpEF), studies have shown that upwards of 50% of patients may have elevated CRP [71–73]. A study comparing 460 patients with HFpEF against HF with reduced ejection fraction (HFrEF) found higher median hsCRP levels in the former (3.6 mg/L [interquartile range 1.8, 7.0] vs. 2.1 mg/L [0.8, 4.7], multivariable adjusted *p* = 0.02), whereas IL-6 and TNF-alpha were not significantly different [74].

Several lines of evidence point to GlycA and GlycB as useful markers for the classification and risk prediction of HF [44, 65, 75]. In an analysis of 6,507 individuals aged 45–84 years in the multi-ethnic MESA study, elevated baseline GlycA level in highest quartile compared with the lowest quartile was associated with increased rates of incident HF during a median follow-up of 14 years [75]. Further analysis of HF subtypes revealed that elevated GlycA was a significant predictor of HFpEF (adjusted HR 2.18 [95% CI, 1.15–4.13]) but not HFrEF (adjusted HR 1.06 [0.63–1.79]) [75]. Those findings may help to link GlycA with aging-related inflammation (inflammaging), myocardial dysfunction, and HFpEF that are associated with old age, diabetes, obesity and multimorbidity [74, 76–80].

A retrospective analysis of cardiac catheterization registry (*n* = 2,996 patients, mean age 63.6 years) suggested that GlycA and hsCRP were independent additive markers for prediction of HF hospitalization in a patient population that had a relatively high prevalence of ischemic heart disease and HF [65]. 65.2% of patients in the cohort had coronary artery disease with > 70% stenosis in at least one vessel, 58% of them had stable angina, 31.8% unstable angina, 10.2% MI and 16.3% HF. Individuals with the uppermost quartile of GlycA level had a 43% increased risk for future major adverse cardiovascular events (MACE) including death, MI, HF hospitalization, and stroke [65]. Among the five MACE endpoints analyzed, HF hospitalization occurred more frequently in patients with above-median than below-median levels of GlycA and hsCRP (HR 2.19, *p* < 0.0001) [65]. However, in a Spanish retrospective cohort (mean age 67.2 years) study, GlycA and GlycB were associated with increased risk for the composite endpoint of all-cause death and HF readmission in non-ischemic but not ischemic HF [44]. Of note, in multivariable adjusted Cox models, GlycB rather than GlycA was more strongly and significantly associated with the risk of HF readmission (HR 2.25 [1.54–3.30] vs. HR 1.09 [0.95–1.25]), occurrence of the composite endpoint (HR 2.13 [1.46–3.13] vs. 1.19 [1.06–1.33]), and recurrent HF hospitalization (HR 1.33 [1.0–1.65] vs. 1.10 [0.94–1.30]) [44].

In a Danish study of 304 T1D patients (median age 62.1 years, duration of diabetes for 33 years, and HbA1c 8.0%), 53.9% of patients were identified as having myocardial dysfunction or subclinical HF [81]. Among them, 94.8% had echocardiographic diastolic dysfunction and elevated N-terminal B-type natriuretic peptide levels. As part of a multi-dimensional predictive model, GlycA was among the most important variable associated with myocardial dysfunction [81]. Indicating impaired relaxation and reduced ventricular compliance (diastolic dysfunction) in patients with T1D, [81] GlycA and GlycB were also associated with increased arterial stiffness (aortic pulse wave velocity vs. GlycA [*r* = 0.55] or GlycB (*r* = 0.42), *p* = 0.001) [82]. Further studies are needed to prospectively determine robustness of their performance in identifying early vascular aging and subclinical HFpEF in patients with established T1D and no prior history of cardiovascular events [82].

## Discriminatory accuracy of GlycA or GlycB from ROC analysis

From the above discussed evidence, it can be appreciated that GlycA and GlycB are good markers for determining the presence or absence of an inflammatory condition. A ROC curve can provide graphical visualization of the discriminatory effectiveness of a given marker to predict or indicate binary outcomes. For patients with T2D and atherogenic dyslipidemia, the GlycA and GlycB variables greatly improved the discrimination between the diseased (either dyslipidemia or T2D) and non-diseased groups [59]. When GlycA and GlycB were added to CRP, the AUC value significantly increased for the classification models between patients with and without dyslipidemia (from 0.54 to 0.79) and between patients with and without diabetes (from 0.55 to 0.76) [59]. Two studies have demonstrated the association of GlycA and GlycB concentrations in patients with rheumatoid arthritis, a disease associated with increased risks for cardiovascular morbidity and mortality. In the study of Fuertes-Martín et al., GlycA in combination with GlycB improved the discriminant capacity of traditional inflammatory variables from AUC 0.70 to 0.79 [23]. In another study, the AUC value for GlycA’s ability to differentiate between patients with low versus moderate to high disease activity was 0.75 [33]. A series of additional studies are needed to broadly evaluate and validate the performance of GlycA and GlycB in different clinical contexts and across populations.

## Impact of interventions on GlycA and GlycB levels

PCSK9 inhibitors (PCSK9i) can effectively reduce LDL-cholesterol levels but biomarkers of inflammation have previously not been shown to change significantly [83]. Using ^1^H-NMR metabolic profiling, Rehues et al. showed that PCSK9i significantly reduced not only levels of total, LDL and non-HDL-cholesterol, and triglycerides but also GlycA, GlycB and apolipoprotein (apo)C-III, and increased apoA-I and HDL-cholesterol levels. Statin therapy has also been shown to lower GlycA levels in separate clinical studies but the magnitude of change was surprisingly modest [84, 85]. A randomized controlled trial on 10-month cardiac telerehabilitation demonstrated improved GlycA (from 840.7 to 758.2 umol/L, *p* = 0.007), GlycB (from 406.5 to 369.9 umol/L, *p* = 0.002) and lipoprotein particle profile, [86] and superiority over center-based cardiac rehabilitation. Further evidence of an intervention having significant impact on GlycA lowering has come from a study of obese patients undergoing bariatric surgery that showed an average of 22% reduction, and a strong association with HDL particle size (*r* = -0.49, *p* < 0.001) that explained 43% of body weight loss [87].

## Limitations and strengths

Clearly, if GlycA and GlycB are to be used as clinical diagnostic tools, they must be thoroughly tested on large cohorts from different populations in a wide range of clinical settings, and meet in vitro diagnostic standards. Moreover, the use of serum versus plasma to establish reference ranges for GlycA and GlycB must be separately considered due to differences in the composition of the biofluids. As above discussed, the resources and facilities required for generating NMR signals of GlycA and GlycB may not be accessible to many clinicians and researchers worldwide. Recently, investigators have used benchtop NMR relaxometry to derive plasma and serum water T_2_ signal that correlates well with inflammatory markers in metabolic syndrome, [88, 89] and may present opportunities for affordable and practical (approaching point-of-care) profiling of early metabolic disorder and risk profiling in the clinic.

In efforts towards maximizing ^1^H-NMR performance and repeatability, a study involving three different laboratories across Europe (Denmark, Germany and The Netherlands) showed that an automated system consisting of the 600 MHz NMR spectrometer, Bruker Avance III, and other associated components of the Bruker IVDr pipeline can achieve very small inter-laboratory variation in profiling lipoprotein particles that even surpasses the acceptable intra-laboratory variation on quality control samples [90]. Comparing methylene and methyl peaks (1.4–0.6 ppm), the study showed that 97.99% of the variance was related to subject differences, whereas 1.62% and 0.39% variance were attributable to sample type (serum versus plasma) and laboratory, respectively.

## Conclusions

Low-grade inflammation of cardiometabolic disease is associated with modification of serum or plasma glycoproteins that can be quantifiable by ^1^H-NMR spectroscopy accurately and reproducibly. Recent studies have provided moderately strong evidence that circulating GlycA and GlycB can extrapolate to early or subclinical cardiometabolic disease phenotypes and predict adverse clinical outcomes. GlycA has been shown to provide independent prediction of incident diabetes and even prediabetes. Its prediction of atherosclerotic cardiovascular disease complications may be superior to hsCRP, pending further prospective studies. Distinct from GlycA, GlycB may differentially inform about heart failure-associated adverse outcomes including rehospitalization that warrant further investigation. Establishing reference levels for GlycA and GlycB in healthy populations of different ages and ethnicity may inform prediction of the onset of cardiometabolic disease, effectiveness of lifestyle intervention, and response to therapy.

### Supplementary Information

**Additional file 1: Supplementary Figure 1.** NMR spectra from the U.S. patent (no. 9792410) of Otvos et al. demonstrating signal peaks of GlycA and GlycB.

## Data Availability

Not applicable. Data used to generate Fig. 1 are available upon request.
