# Supplementary material for: Towards clinical application of GlycA and GlycB for early detection of inflammation associated with (pre)diabetes and cardiovascular disease: recent evidence and updates
Source: J Inflamm (Lond). 2023 Oct 9;20:32. doi: 10.1186/s12950-023-00358-7 (PMC10563214; doi:10.1186/s12950-023-00358-7)
Supplement: Supplementary file 1 — Additional file 1: Supplementary Figure 1. NMR spectra from the U.S. patent (no. 9792410) of Otvos et al. demonstrating signal peaks of GlycA and GlycB. [file 12950_2023_358_MOESM1_ESM.docx]

**Supplementary Figure 1**. NMR spectra from the U.S. patent (no. 9792410) of Otvos et al. demonstrating signal peaks of GlycA and GlycB.


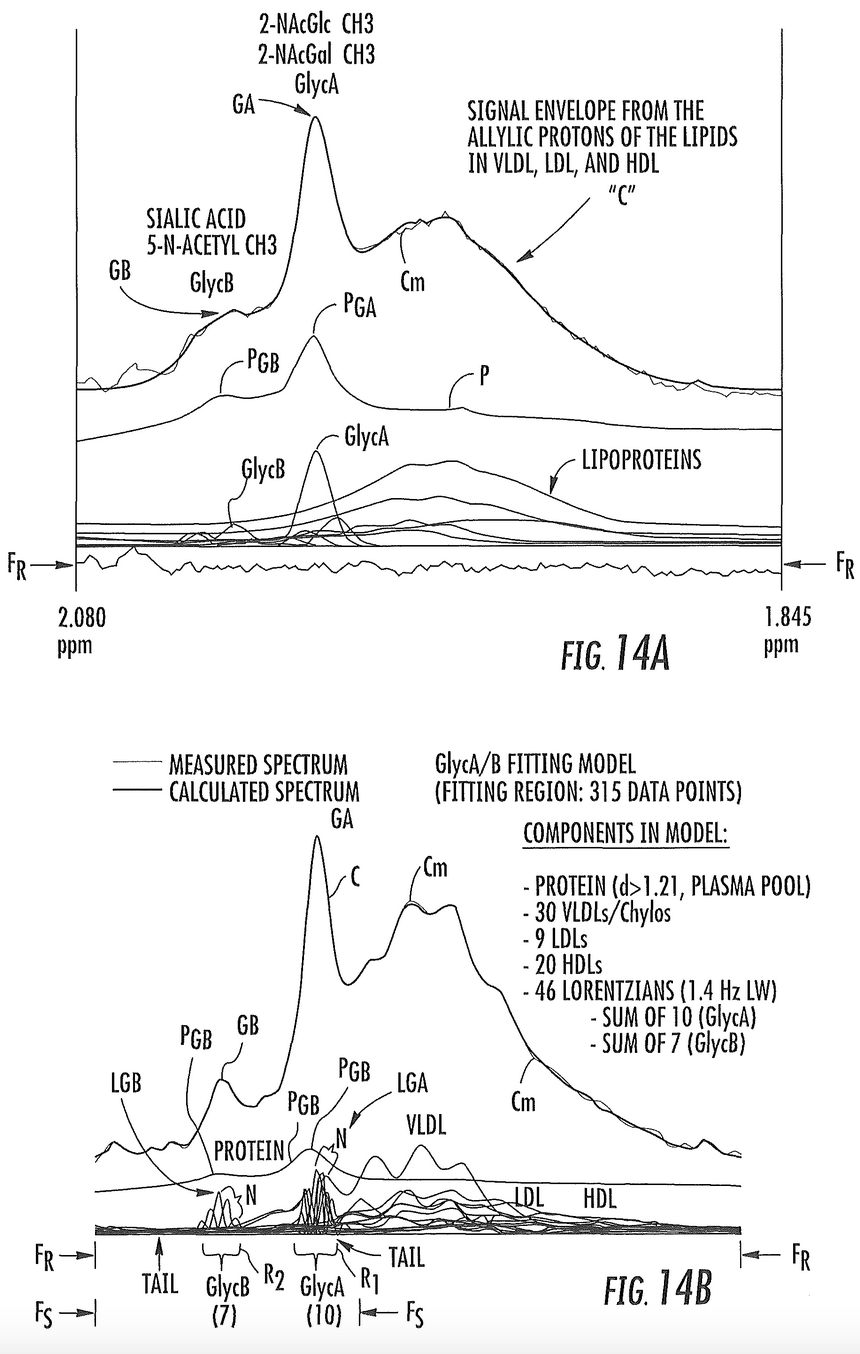


Reference:

Otvos JD, Shalaurova IY, Bennett DW, Wolak-Dinsmore JE, O'Connell TM, Mercier K. Multi-parameter Diabetes Risk Evaluations. Patent number 9792410. Published by U.S Patent Office (publication number 20150149095) on Oct 17, 2017.
